# Cancer

**Published:** 1887-11

**Authors:** 


### CANCER.

This disease appears to be largely on the increase. From the report of the Registrar-General, we learn there were 80,049 deaths in England from this cause during the ten years, from 1860 to 1869, an annual increase of two hundred and forty-eight. From 1870 to 1872 the annual increase was three hundred and twenty-seven. Though we have no statistics at hand, medical practitioners are generally inclined to admit that this disease is alarmingly on the increase in the United States.

It seems to be more prevalent in the heated and intense life of cities than in the country ; among the rich and well-to-do than among the poor. According to French authority, the proportion of cancer in France among the wealthy classes is about one hundred and six in one thousand ; among the poorer classes seventy two in one thousand, ten per cent. of the former, and seven per cent. of the latter. It seems to be a morbid growth upon civilization, as it is rarely found among savage men. Like the scale and other fruit pests that prey upon our orchards and vineyards, the shadow of this baleful parasitic growth hovers around the homes of ease, luxury and high living, rather than pioneer cabins, the camps and huts of miners, loggers, hunters and fishermen. Observation shows that it is fond of localities. It is said to be wholly unknown in the Faroe islands. In Iceland in one year, there were only thirty seven cases out of fifty thousand inhabitants. Considering the geographical extent of England, it is more prevalent, malignant and fatal there than elsewhere, and among English-speaking people than any other race. It is to be found more largely among women than men, and more among those who have been mothers. It is most likely to appear between the ages of thirty-five and sixty. It stands almost alone as a disease that increases with our excited life and prosperity, and while health-laws and improved sanitation are raising the standard of health, this hideous and loathsome malady rests as a blot upon our vital statistics.

As far as known cancer does not arise from any micro-organisms or germs, and consequently is not infectious or contagious. It cannot be caught nor given. But if it be true that cancerous persons do not propagate the disease, it is almost certain they beget consumptive children ; and consumption is always a result of debility, want of general health, whatever may be the immediate cause. Still, cancer often attacks persons in the most vigorous health, and consequently is hard to classify in terms of causation.

It is a sad commentary on our boasted healing art, with its rich heritage of more than a thousand years, that this disease is still classed among the incurables. Many of our most learned and widely-experienced physicians confess they have no faith in any known remedy, and believe that all that can be done is to mercifully let the wretched sufferer as softly into the grave as possible. There are a few who still think that electricity may furnish some alleviation, but as a curative agent, it has but few followers. It seems to be a melancholy concurrence among medical men that no confidence can be placed in medication, and that, as long as the etiology of the disease is wrapped in mystery, it is a waste of time to discuss its therapeutics. The knife is now about the only accepted remedy with the profession throughout the world, but even that it is admitted can only avail in the incipiency of the disease, which is liable to break out again. It is always a local disease at the start, but spreads by means of blood-vessels and lymphatics, first to the nearest gland, then more distant, and when this occurs it is no longer local but constitutional, and the surgeon is powerless. At the start, cancer seems closely allied with the tumor family, and has usually become constitutional before it unmasks itself. Once in the blood it is hopelessly beyond all surgical eradication.

There is an array of evidence placing beyond all dubitation, the fact that there are specialists who have, apparently, a successful cancer record unknown to the medical world.

Why the medical faculty should evince a disinclination to add these facts to their pathological knowledge, is only another illustration of the many in history, of the human mind being hampered by theory, warped by prejudice and manacled by absurdly limiting and restraining rules.

These learned and diplomaed gentlemen should remember that it is no strange thing for nature to pass by academies and laboratories of science, and place the crown of discovery upon an obscure and unscientific head.

But it is said that these particular specialists use secret remedies, and advertise their business. This is most likely the real point where the shoe pinches. It would not be difficult to show that many practitioners of the regular school, whose names stand high on the roll of fame, have used secret remedies that were not revealed till near the close of life. These learned men practice their art to make money, and as human nature is all off the same piece, we have serious doubts if there is one of them, who, if in possession of a secret remedy, would not keep it under cover until he had reaped a rich harvest from his skill and experience. But this is a question of life or death, and the doctor who admits that he is powerless in the presence of cancer, and lets his patient die when he has indubitable proof, or even presumptive proof, that there is a remedy not far away, and will not use it nor recommend its use because there is a little mystery hanging over its composition, would seem to the outside world as committing a crime little short of murder.

